# miR-367-3p靶向ZEB2抑制NSCLC细胞增殖、迁移和侵袭生物学功能研究

**DOI:** 10.3779/j.issn.1009-3419.2022.101.49

**Published:** 2022-11-20

**Authors:** 延军 苏, 华 张

**Affiliations:** 300060 天津，天津医科大学肿瘤医院肺部肿瘤科；国家肿瘤临床医学中心；天津市“肿瘤防治”重点实验室；天津市恶性肿瘤临床医学研究中心；天津市肺癌中心 Department of Lung Cancer Surgery, Tianjin Cancer Hospital Affiliated to Tianjin Medical University; National Cancer Clinical Medical Center; Tianjin Key Laboratory of Cancer Prevention and Treatment; Tianjin Cancer Clinical Medicine Research Center; Tianjin Lung Cancer Center, Tianjin 300060, China

**Keywords:** 肺肿瘤, miR-367-3p, 侵袭, 转移, Lung neoplasms, miR-367-3p, Invasion, Metastasis

## Abstract

**背景与目的:**

微小RNA在肺癌的发生发展及生物学表型中发挥重要作用。探讨miR-367-3p在非小细胞肺癌（non-small cell lung cancer, NSCLC）患者中的表达及其对NSCLC细胞增殖、迁移和侵袭的影响。

**方法:**

选取我院收治并手术治疗的NSCLC患者22例（其中腺癌13例，鳞癌9例），术中留取患者癌组织、癌旁组织及外周血5 mL，同时选取健康查体患者22例，留取外周血5 mL。采用Real-time PCR法检测NSCLC患者癌组织、癌旁组织、外周血及健康查体者外周血中miR-367-3p的相对表达水平。培养肺癌细胞株（A549）与正常支气管上皮细胞（bronchial epithelium transformed with Ad12-SV40 2B, BEAS-2B）并检测细胞株中miR-367-3p表达水平。转染外源性miR-367-3p后采用MTT和Transwell实验观察转染前后A549细胞增殖和侵袭能力变化。采用生物信息分析miR-367-3p下游靶基因，采用Real-time PCR和Western blot检测转染外源性miR-367-3p前后锌指e-box-binding同源盒2（Zinc finger E-box binding homeobox 2, ZEB2）表达水平。

**结果:**

22例NSCLC患者，癌组织中miR-367-3p相对表达水平显著低于癌旁组织（*P* < 0.05），同时健康查体者血清中miR-367-3p水平显著高于NSCLC患者（*P* < 0.05）；分别以组织和血清中miR-367-3p的相对表达水平为参考，诊断NSCLC受试者工作特征（receiver operating characteristic, ROC）曲线下面积分别为0.95（95%CI: 0.89-1.00）和0.85（95%CI: 0.74-0.97）；肺癌细胞株A549转染外源性miR-367-3p后，细胞增殖、迁移能力显著降低（*P* < 0.05）；生物信息预测miR-367-3p下游靶基因为*ZEB2*，上调miR-367-3p表达后，*ZEB2*基因表达水平降低（*P* < 0.05）。癌症基因组图谱（The Cancer Genome Atlas, TCGA）数据分析ZEB2表达水平与NSCLC患者预后关系显示，ZEB2高表达组NSCLC患者总生存（overall survival, OS）和无疾病进展生存（disease free survival, DFS）与低表达组NSCLC差异无统计学意义（*P* > 0.05），但高表达组显示出了OS及DFS降低的趋势。

**结论:**

miR-367-3p在NSCLC患者中表达下调，并通过靶向*ZEB2*基因参与NSCLC的增殖和侵袭生物学过程。

## Abstract (en)

**Background and objective:**

microRNAs play an important role in the development and biological phenotype of lung cancer. The present study was to investigate miR-367-3p level in non-small cell lung cancer (NSCLC) patients and its biological function of NSCLC cells.

**Methods:**

Twenty-two patients with NSCLC (13 cases of adenocarcinoma and 9 cases of squamous carcinoma) admitted to our hospital and treated by surgery were included. During the operation, cancer tissue, paracancerous tissue and 5 mL peripheral blood were collected. Meanwhile, 22 healthy controls were selected and 5 mL peripheral blood was taken. Real-time PCR was applied to detected the expression of miR-367-3p in cancer tissues, peripheral blood of patients with NSCLC and healthy controls. miR-367-3p was detected in lung cancer cell lines (A549) and normal bronchial epithelial cells (BEAS-2B). The proliferation and invasion ability of A549 cells before and after infection were detected by MTT and Transwell assay after transfection with exogenous miR-367-3p. The downstream target gene of miR-367-3p was analyzed by bioinformatics. Zinc finger E-box binding homeobox 2 (ZEB2) was detected by Real-time PCR and Western blot.

**Results:**

miR-367-3p in cancer tissues of 22 NSCLC patients was lower than corresponding normal tissues (*P* < 0.05), and the serum miR-367-3p level in healthy subjects was higher than NSCLC subjects (*P* < 0.05). The area under the receiver operating characteristic (ROC) curve of NSCLC was 0.95 (95%CI: 0.89-1.00) and 0.85 (95%CI: 0.74-0.97) respectively; The proliferation and migration ability of lung cancer cell line A549 transfected with exogenous miR-367-3p decreased significantly (*P* < 0.05); Bioinformatics predicted that the downstream target of miR-367-3p was ZEB2 and up-regulating miR-367-3p expression, *ZEB2* gene was decreased (*P* < 0.05). The Cancer Genome Atlas (TCGA) data analysis showed that there was no significant difference in overall survival (OS) and disease free survival (DFS) between ZEB2 high expression group and low expression group (*P* > 0.05). ZEB2 expression was positively correlated with infiltration of B lymphocytes (*r*=0.32, *P* < 005), CD8^+^ T cells (*r*=0.44, *P* < 005), CD4^+^ T cells (*r*=0.46, *P* < 005), macrophages (*r*=0.65, *P* < 005), neutrophils (*r*=0.73, *P* < 005) and dendritic cells (*r*=0.71, *P* < 005) in NSCLC.

**Conclusion:**

The expression of miR-367-3p is down regulated in NSCLC patients and participates in the biological process of proliferation and invasion of NSCLC by targeting *ZEB2* gene.

肺癌是临床上最为常见的恶性肿瘤，而非小细胞肺癌（non-small cell lung cancer, NSCLC）占整体肺癌发病率的80%左右^[1]^。流行病学研究^[2, 3]^显示，肺癌在男性发病率为第一位，女性为第二位。在所有肿瘤导致的死亡原因中，无论男性和女性，肺癌均为第一位的肿瘤相关死亡原因^[2]^。然而，NSCLC的确切发病原因、侵袭转移等生物学行为及其分子机制并未完全阐明。然而，越来越多的研究^[4-6]^显示微小RNA（microRNA）在肿瘤的发生发展、侵袭转移和表观遗传领域发挥重要的作用。

miR-367-3p的前体为hsa-miR-367，定位于人4号染色体chr4:112647874-112647941^[7]^。近年来研究^[8, 9]^显示miR-367-3p在人多种肿瘤中存在差异表达，并参与肿瘤细胞的恶性表型。然而，miR-367-3p在NSCLC中的研究报道较少，其生物学功能及相关分子机制不明。

## 1 资料与方法

### 1.1 肺癌患者及健康查体者

选取22例NSCLC患者的组织样本和配对正常肺组织为研究对象。同时选取22例健康查体者，留取外周静脉血。NSCLC患者纳入标准：NSCLC明确病理学诊断且接受手术治疗者。相关影像学检查除外远处转移者。排除标准：小细胞肺癌患者；接受新辅助放化疗或免疫治疗者。切除的肺癌组织均经过天津医科大学肿瘤医院病理科验证为NSCLC。所有肺癌组织存放在液氮中冷冻，并储存于-80 ℃冰箱，直到RNA提取。22例NSCLC患者年龄46岁-74岁，平均年龄（59.3±16.2）岁，男性14例，女性8例；腺癌13例，鳞癌9例。

### 1.2 Real-time PCR试验

采用RNA提取试剂盒，提取上述组织和血液学标本中的总RNA，采用M-MLV逆转录试剂盒合成cDNA。采用Real-time PCR法检测miR-367-3p和锌指e-box-binding同源盒2（Zinc finger E-box binding homeobox 2, ZEB2）相对表达水平，引物序列见表 1。反应体系和条件如下，按20 μL体系进行基因表达水平检测：SYBR premix 10 μL，GAPDH上游引物或miR-367-3p上游引物1 μL；GAPDH下游引物或miR-367-3p下游引物1 μL；cDNA 1 μL，灭菌去离子水8 μL。扩增条件为：95 ℃、5 min；95 ℃、30 s，58 ℃、30 s，72 ℃、30 s，循环40次；72 ℃、10 min。以2^-ΔΔCt^（Ct为循环阈值）法来表示Real-time PCR结果的相对表达量。

**表 1:** miR-367-3p表达水平检测Real-time PCR引物 Primer of miR-367-3p detected by Real-time PCR

Primer	Sequence (5’-3’)
F: miR-367-3	AGTGCAGGGTCCGAGGTATT
R: miR-367-3p	CGACGAATTGCACTTTAGC
F: ZEB2	ACTTTTCCTGCCCTCTCTGT
R: ZEB2	TTGCGATTACCTGCTCCTT
F: GAPDH	GAAGGTGAAGGTCGGAGTC
R: GAPDH	GGGTGGAATCATATTGGAAC

### 1.3 转染外源性miR-367-3p

培养A549细胞，应用RMPI-1640+10%FBS完全培养基，置于37 ℃培养。细胞传代两次至生长状态良好后，消化细胞形成细胞悬液，包含细胞数为5×10^5^个，轻轻摇动6孔板，细胞混合均匀后，继续培养。待生长3 d后，开始进行转染。miR-367-3p模拟物及无义对照序列（miR-NC）均委托苏州吉玛基因股份有限公司合成。采用Lipofectamine 3000转染试剂进行转染，转染方法按试剂盒说明进行。转染设置4组：空白对照组（细胞未经处理）、Mock组（细胞中仅加入转染试剂Lipofectamine 3000模拟转染过程）、NC组（细胞转染miR-NC无义序列，作为miR-367-3p阴性对照）、实验组（细胞转染miR-367-3p模拟物）。

### 1.4 MTT试验

消化培养瓶中的A549细胞，吹散并进行细胞计数，调整细胞浓度为1×10^4^个/mL，向96孔板中每孔加入200 μL细胞悬液，即2,000个细胞/孔。待细胞密度至70%，进行细胞转染。转染设置4组，每组设置6个副孔，分别为空白对照组、Mock、NC和实验组。细胞转染6 h后，更换新的完全培养基，继续培养。培养分别于转染0 h、24 h、48 h、72 h、96 h后，弃去各孔培养基，PBS清洗3次，加入200 μL新鲜完全培养基，并加入5 mg/mL MTT溶液20 μL，继续将培养板置于37 ℃、5%CO_2_培养箱中培养4 h，4 h后弃去上清，每孔加入150 μL DMSO，结晶物溶解后，测量OD490 nm吸光值，上机时设置调零孔。以时间梯度为横坐标（X轴），吸光值为纵坐标（Y轴）绘制曲线图。

### 1.5 Transwell试验

消化培养瓶中的A549细胞，吹散并进行细胞计数，细胞浓度为5×10^5^个/mL，向24孔Transwell板中上室每孔加入100 μL细胞悬液，即5×10^4^个细胞/孔，下室加入600 μL完全培养基。待细胞密度至70%，进行细胞转染。细胞转染6 h后，更换新的完全培养基，置于37 ℃、5%CO_2_培养箱中继续培养。培养48 h后，弃去上室培养基，刮去上室膜上贴壁细胞，PBS清洗3次，甲醇固定30 min，将小室晾干后，用0.1%结晶紫染色20 min，PBS清洗3次，显微镜下随机观察9个视野细胞，并计数。

### 1.6 Western blot试验

提取细胞中的总蛋白，分别进行制胶、垂直电泳、转膜、封闭等操作。然后进行一抗孵育：配制一抗溶液各4 mL并加入孵育盒中，4 ℃过夜。二抗孵育：回收一抗溶液，配制辣根酶标记的山羊抗兔和山羊抗小鼠的二抗溶液，注入孵育盒中，于常温慢摇1 h。孵育结束后漂洗NC膜3次，曝光及蛋白条带分析。

### 1.7 生物信息分析

microRNA靶基因预测数据库Targetscan（http://www.targetscan.org/vert_72/）和StarBase（http://starbase.sysu.edu.cn/starbase2/）对miR-367-3p下游靶基因进行预测。癌症基因组图谱（The Cancer Genome Atlas, TCGA）数据库中（https://www.cancer.gov/），根据ZEB2中位表达水平，将NSCLC患者分为ZEB2高表达组（肿瘤组织中ZEB2表达≥中位表达水平）和低表达组，采用*Log-rank*检验比较高低标的组NSCLC患者无疾病进展生存（disease free survival, DFS）和总生存（overall survival, OS）有无差异。TIMER数据库（https://cistrome.shinyapps.io/timer/）分析ZEB2基于表达与NSCLC免疫细胞浸润情况。

## 2 结果

### 2.1 miR-367-3p在NSCLC患者及健康者中的表达水平比较

应用RT-PCR方法检测22例患者癌组织和正常组织中的miR-367-3p表达水平，结果表明癌组织中miR-367-3p表达水平是明显低于正常组织（图 1A），差异具有统计学意义（*P* < 0.05），说明肺组织中miR-367-3p的低表达可能与肺癌发生具有一定的相关性。22例肺癌患者血清和22例健康人血清中的miR-367-3p表达水平表明肺癌患者血清中miR-367-3p表达水平明显低于健康人（图 1B），差异具有统计学意义（*P* < 0.05），说明外周血清中miR-367-3p的低表达与肺癌发生具有一定的相关性。

**图 1:**
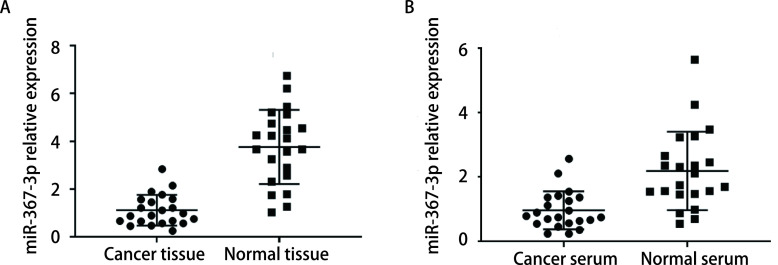
miR-367-3p在组织和血清中表达水平比较。A：miR-367-3p在NSCLC患者癌组织和癌旁组织中的表达比较；B：miR-367-3p在NSCLC患者和健康查体者血清中的比较。 Comparison of miR-367-3p expression in tissues and serum. A: comparison of miR-367-3p expression in cancer tissues and adjacent tissues of NSCLC patients; B: comparison of miR-367-3p expression in serum of NSCLC patients and healthy subjects. NSCLC: non-small cell lung cancer.

### 2.2 miR-367-3p诊断NSCLC的临床价值

分别以组织和血清中miR-367-3p的相对表达水平为参考，诊断NSCLC受试者工作特征（receiver operating characteristic, ROC）曲线下面积分别为0.95（95%CI: 0.89-1.00）和0.85（95%CI: 0.74-0.97），见图 2。

**图 2:**
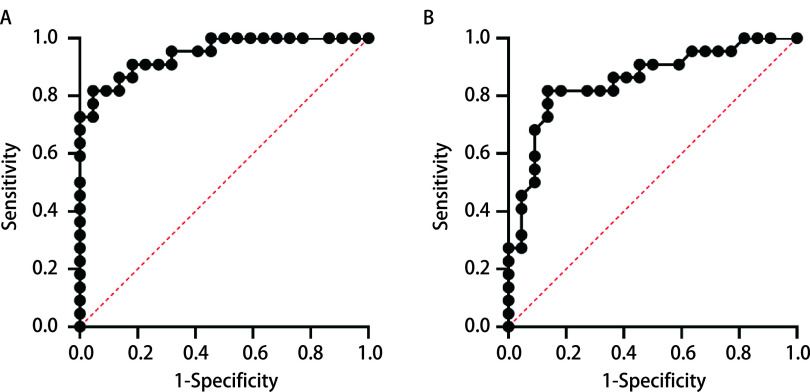
miR-367-3p诊断NSCLC的ROC曲线。A: 组织; B: 血清。 ROC curve of miR-367-3p in diagnosis of NSCLC. A: tissue; B: serum. ROC: receiver operating characteristic.

### 2.3 miR-367-3p在细胞系中的表达水平分析

miR-367-3p在A549细胞系中的表达水平显著低于正常支气管上皮细胞BEAS-2B（*P* < 0.05）（图 3A）；转染外源性miR-367-3p后，实验组miR-367-3p mRNA表达水平明显高于空白对照组、Mock组和NC组（图 3B）（*P* < 0.05）。

**图 3:**
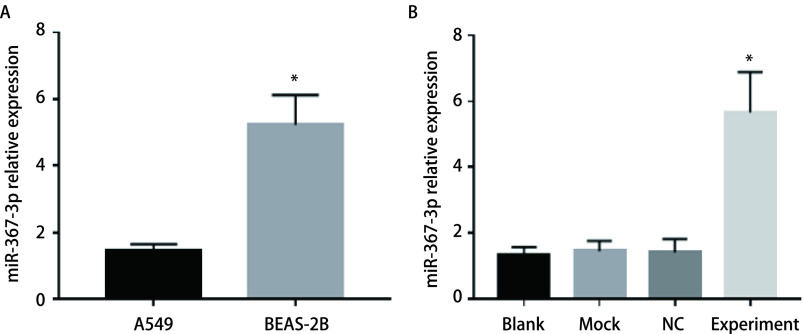
miR-367-3p在细胞系中的表达水平。A: miR-367-3p在肺癌细胞系A549和正常支气管上皮细胞BEAS-2B中的表达；B: miR-367-3p在经不同处理的A549细胞中的表达。 Expression level of miR-367-3p in cell lines. A: Expression of miR-367-3p in lung cancer cell line A549 and normal bronchial epithelial cell line BEAS-2B; B: Expression of miR-367-3p in A549 cells after different treatments. ^*^P < 0.05.

### 2.4 上调miR-367-3p表达后肺癌细胞增殖能力减低

MTT结果表明，实验组细胞增殖能力明显低于空白对照组、Mock组和NC组（图 4），差异具有统计学意义（*P* < 0.05）。说明miR-367-3p模拟物可明显抑制A549肺癌细胞的增殖能力，其抑制能力与时间梯度呈正相关。

**图 4:**
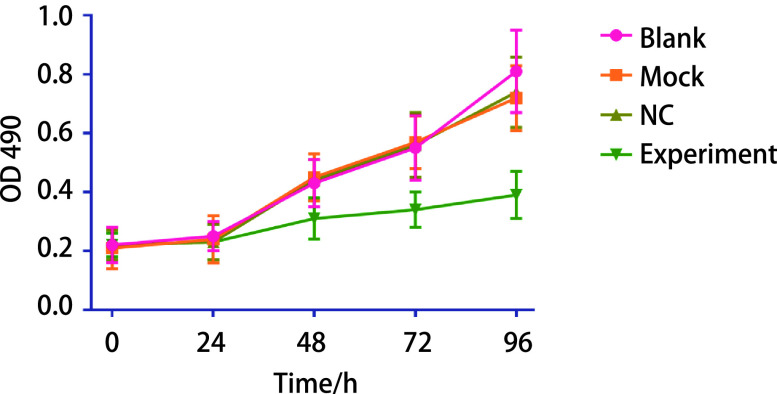
过表达miR-367-3p后肺癌细胞增殖能力变化 Changes in proliferation of lung cancer cells after overexpression of miR-367-3p

### 2.5 上调miR-367-3p后肺癌细胞侵袭能力减低

Transwell结果表明，实验组A549细胞迁移数量明显低于空白对照组、Mock组和NC组（图 5），差异具有统计学意义（*P* < 0.05），说明转染miR-367-3p模拟物可明显抑制A549细胞的迁移能力。

**图 5:**
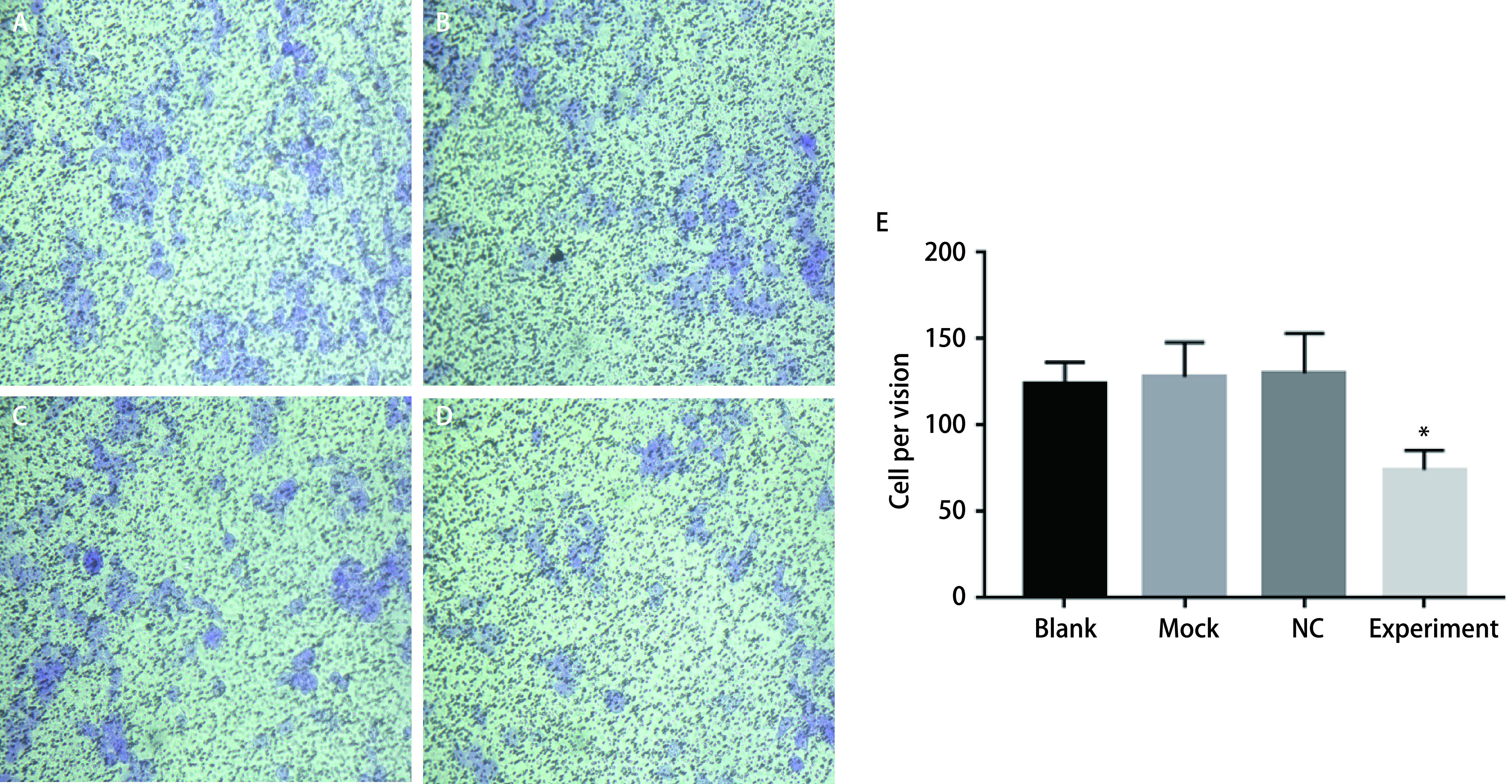
过表达miR-367-3p后肺癌细胞侵袭能力。A：空白组；B：Mock组；C：NC组；D：试验组；E：侵袭能力柱状图。 Invasion ability of lung cancer cells after overexpression of miR-367-3p. A: blank group; B: Mock group; C: NC group; D: experimental group; E: invasiveness histogram.

### 2.6 miR-367-3p靶基因预测及表达水平分析

microRNA靶基因预测数据库Targetscan和StarBase对miR-367-3p下游靶基因进行预测，结果显示*ZEB2*基因于miR-367-3p的3’UTR特异性结合，提示*ZEB2*可能为miR-367-3p的靶基因。实验组ZEB2蛋白表达水平明显高于空白对照组、Mock组和NC组（图 6），差异具有统计学意义（*P* < 0.05），说明miR-367-3p模拟物可调节ZEB2蛋白的表达水平。

**图 6:**
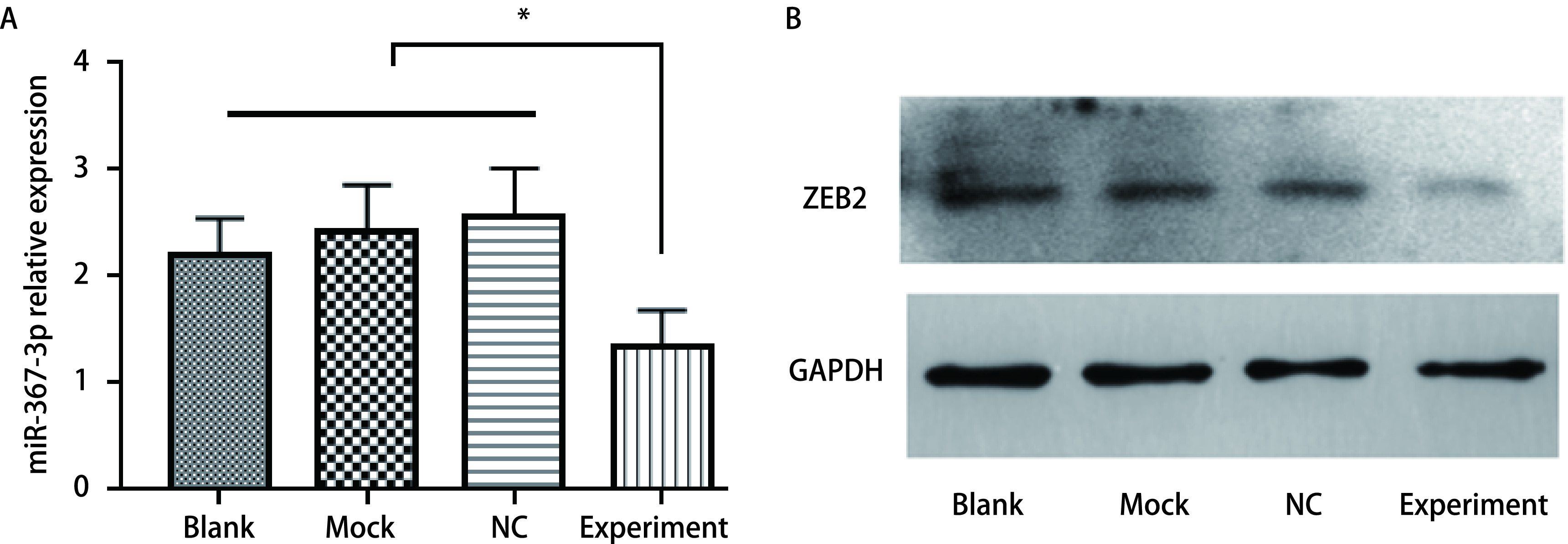
miR-367-3p靶基因*ZEB2*表达水平分析。A：ZEB2在不同处理A549细胞中的mRNA表达情况；B：ZEB2在不同处理A549细胞中的蛋白表达情况。 Expression of miR-367-3p target gene *ZEB2*. A: mRNA expression of ZEB2 in A549 cells in different groups; B: Protein expression of ZEB2 in A549 cells in different groups. ^*^P < 0.05.

### 2.7 miR-367-3p靶基因*ZEB2*表达与NSCLC患者预后

TCGA数据库中（https://www.cancer.gov/），根据ZEB2中位表达水平，将NSCLC患者分为ZEB2高表达组（肿瘤组织中ZEB2表达≥中位表达水平）和低表达组，采用*Log-rank*检验比较高低标的组NSCLC患者DFS和OS有无差异。结果显示，ZEB2高表达组NSCLC患者OS和DFS与低表达组NSCLC差异无统计学意义（*P* > 0.05），但高表达组显示出了OS及DFS降低的趋势，图 7。

**图 7:**
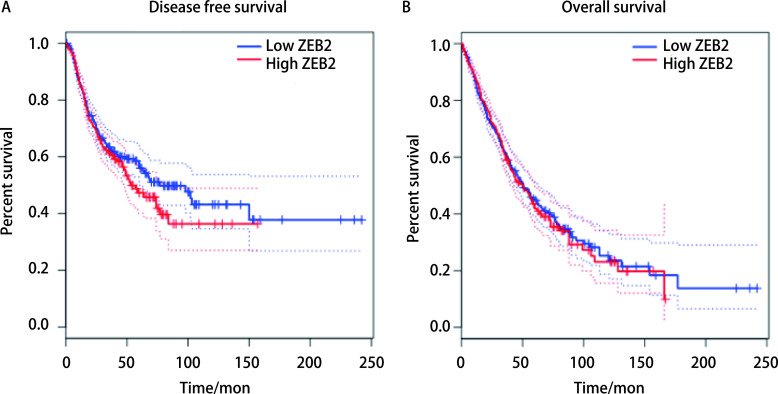
miR-367-3p靶基因*ZEB2*表达与NSCLC患者预后。A：高低表达组患者DFS比较；B：高低表达组患者OS比较。 miR-367-3p target gene *ZEB2* expression and prognosis of NSCLC patients. A: DFS comparison of high and low expression group; B: OS comparison of high and low expression group. OS: overall survival; DFS: disease free survival.

### 2.8 ZEB2表达与免疫细胞浸润

TIMER数据库中分析ZEB2基于表达与NSCLC免疫细胞浸润情况，ZEB2表达与B淋巴细胞（*r*=0.32, *P* < 0.05）、CD8^+^ T细胞（*r*=0.44, *P* < 0.05）CD4^+^ T细胞（*r*=0.46, *P* < 0.05）、巨噬细胞（*r*=0.65, *P* < 0.05）、中性粒细胞（*r*=0.73, *P* < 0.05）和树突状细胞（*r*=0.71, *P* < 0.05）浸润均存在正向关系。

## 3 讨论

本研究采用生物信息分析联合细胞分子实验探讨微小RNA在肺癌的发生发展及生物学表型中发挥重要作用。分析miR-367-3p在NSCLC患者中的表达及其对NSCLC细胞增殖、迁移和侵袭的影响。研究中我们通过检测NSCLC患者及健康查体者癌组织、癌旁肺组织、血清中miR-367-3p的表达水平发现，NSCLC患者癌组织miR-367-3p水平显著低于癌旁组织，而NSCLC患者血清中miR-367-3p水平显著低于健康查体者。进一步细胞系中也发现，miR-367-3p在肺癌细胞系A549中的表达水平显著低于正常支气管上皮细胞。上述结果在人体组织、血液及细胞系中均证实miR-367-3p在NSCLC中表达下调。同时已发表的文献也证实，在大多数肿瘤中，miR-367-3p表达下降，这些肿瘤包括胃癌、宫颈癌和睾丸生殖细胞肿瘤等^[10-12]^。

进一步的生物学功能研究显示，上调肺癌细胞株A549中miR-367-3p的表达后，细胞的增殖和迁移能力均明显降低。提示miR-367-3p可能通过参与细胞增殖和侵袭相关信号通路，影响A549细胞的增殖和迁移能力改变。国内杜宁等^[13]^探讨miR-367-3p在NSCLC组织中的表达及对细胞功能的影响，通过检测NSCLC组织中miR-367-3p的水平，也证实其在癌组织中的表达水平明显高于癌旁组织，与本研究结果一致。同时研究还发现，miR-367-3p可能通过靶向下游FBXW7的表达，而影响细胞增殖和细胞周期^[14]^。在本研究中，我们通过生物信息分析，预测了miR-367-3p与*ZEB2*基因mRNA能特异性结合，提示*ZEB2*为其下游靶基因。进一步的分析显示，上调miR-367-3p表达后，A549细胞的增殖和迁移能力明显降低，而上调miR-367-3p后*ZEB2*基因mRNA和蛋白表达水平均明显降低，进一步提示miR-367-3p可能通过靶向*ZEB2*基因表达而发挥生物学功能。

同时，我们选取TCGA数据库中NSCLC患者的预后资料作为研究对象，分析*ZEB2*基因表达水平与NSCLC患者预后的关系，结果虽然显示ZEB2高表达组NSCLC患者OS和DFS与低表达组差异无统计学意义（*P* > 0.05），但高表达组显示出了OS及DFS降低的趋势。因此，我们研究结果显示，miR-367-3p在NSCLC患者中表达下调，并通过靶向*ZEB2*基因参与NSCLC的增殖和侵袭生物学过程，提示miR-367-3p可能成为NSCLC患者靶向治疗和靶向药物研发的新靶点^[15]^。
